# The effect of full‐limb flexion tests on static and dynamic muscle activity and locomotion asymmetry in owner‐sound horses

**DOI:** 10.1111/evj.70130

**Published:** 2025-12-02

**Authors:** Marijke Jonkhart, Filipe M. Serra Bragança, Ineke H. Smit, Harold Brommer, Jozef J. M. Suskens

**Affiliations:** ^1^ Department of Clinical Sciences, Faculty of Veterinary Medicine Utrecht University Utrecht The Netherlands; ^2^ Sleip AI Stockholm Sweden

**Keywords:** electromyography, flexion tests, gait analysis, horse, locomotion symmetry, muscle activity

## Abstract

**Background:**

Flexion tests are commonly used in equine locomotion examinations to identify underlying locomotor issues, yet their neuromuscular effects remain poorly understood. Response variability raises concerns about their clinical value in lameness assessments and pre‐purchase evaluations.

**Objectives:**

Primarily, to investigate the effect of full‐limb flexion tests on static (flexed position) and dynamic (subsequent trot‐up) muscle activity. Secondarily, to assess their effect on locomotion asymmetry during trotting.

**Study Design:**

In vivo experiments.

**Methods:**

Sixteen warmblood horses were randomly assigned to one of four groups (*n* = 4) for surface electromyography of selected limbs. Forelimb region muscles included Splenius muscle, Triceps brachii caput longus muscle, Longissimus dorsi muscle, Extensor digitorum communis muscle, and Ulnaris lateralis muscle, while hindlimb region muscles included Gluteus medius muscle, Biceps femoris muscle, Semitendinosus muscle, Extensor digitorum longus muscle, and Flexor digitorum profundus muscle. Electromyographic data were side‐normalised, and kinematic data from inertial measurement units assessed asymmetry in head, withers, and pelvis. Each limb underwent a 60‐s full‐limb flexion test, followed by straight‐line trotting. Muscle activity in static and dynamic phases was compared to baseline using one‐way repeated measures ANOVA, and two‐way repeated measures ANOVA with statistical parametric mapping, respectively. Kinematic variables were compared using a linear mixed‐effect model.

**Results:**

No significant differences in normalised muscle activity were observed in fore‐ or hindlimb region muscles during flexion or subsequent trotting. However, pelvic displacement increased during the suspension phase of the ipsilateral limb, with significant vertical displacement (MaxDiff) after left (8.4 mm ± 2.4 SE [standard error], *p* = 0.002) and right (8.1 mm ± 2.4 SE, *p* = 0.004) hindlimb flexion test.

**Main Limitations:**

Only full‐limb flexion tests were investigated, limiting generalisability to distal or carpal/tarsal tests. Flexion force was not standardised.

**Conclusions:**

We found no evidence that flexion tests affect equine neuromuscular functioning in the muscles we investigated.

## 1 INTRODUCTION

Flexion tests have been an integral part of clinical lameness examinations and prepurchase evaluations. However, their value has been debated for years.^1^, ^2^, ^3^ A flexion test involves imposing a specific posture on a limb, with one or more joints flexed for a period commonly between 30 and 60 s. It is assumed that by stretching and compressing muscle and connective tissue (e.g., ligaments and joint capsule), temporary vascular restriction occurs, together with the activation of pain receptors.^4^ Therefore, flexion tests are used as provocation tests to induce a temporary, intensified localised pain response, potentially associated with orthopaedic pathology. However, veterinarians perform flexion tests with varying durations, applied force, and number of joints flexed.^5^ The absence of a consensus highlights the limited understanding of the physiological response, which is essential for considering its diagnostic value in daily practice.

Equine veterinarians spend up to 40% of their time conducting clinical lameness examinations, as lameness is a frequent orthopaedic health issue among horses.^6^, ^7^ Flexion tests can cause (increased) locomotion asymmetry, suggesting some sort of movement impairment. Identifying compensatory mechanisms such as locomotion asymmetry could aid in clinical decision‐making during lameness diagnosis, providing an accessible and cost‐effective tool for equine veterinarians.^8^, ^9^ A significant increase in the upward movement of the pelvis segment can be measured when applying a hindlimb flexion test (i.e., ipsilateral MaxDiff).^9^, ^10^ This response diminishes gradually over the first 10 strides of subsequent trotting.^10^ However, the underlying mechanism of this asymmetry remains poorly understood.^8^

Locomotion asymmetry may be attributed to changes in neuromuscular activation. Alterations in muscle activity amplitude and duration of muscle activation have been measured in response to induced forelimb and hindlimb lameness, affecting both appendicular and spinal muscles.^11^, ^12^ To date, no studies have examined muscle activity during the flexion test itself or the impact on muscle activity during subsequent locomotion (e.g., trot‐up). Furthermore, limited research has investigated the anatomical structures affected by the flexion test. Although the flexion test is primarily applied on the limb of interest, veterinarians often report a positive effect on the contralateral limb as well.^4^ This suggests that flexion tests may not only affect the flexed limb but also influence the contralateral weight‐bearing limb.

This study aimed to investigate the effect of full‐limb flexion tests on muscle activity and locomotion asymmetry in owner‐sound horses (i.e., horses considered lame‐free by their owner), mimicking common practice in pre‐purchase exams. We also aimed to add to the understanding of the neuromuscular responses underlying flexion tests to aid equine lameness diagnosis. We hypothesised that flexion tests would induce significant changes in muscle activity in the contralateral supporting limb during the full‐limb flexion phase and in the flexed limb during subsequent trotting. This neuromuscular response was expected to correspond with significant alterations in locomotion asymmetry, reflecting limb unloading strategies.

## 2 MATERIALS AND METHODS

### 2.1 Animals

Eight adult horses per front‐ and hind‐side were deemed sufficient for adequate power, based on previous locomotion studies on surface electromyography (EMG) and movement symmetry.^11^, ^13^ Sixteen owner‐sound warmblood horses were recruited from the institution's own educational horses (*n* = 3) and through institutional and personal networks (*n* = 13). Interested horse owners received information regarding the study protocol and signed informed consent prior to participation. The inclusion criterion for participation was a minimal measurable objective asymmetry. For this, a smartphone application (Sleip) was used while the horse trotted up and down twice on a 30‐m straight and hard surfaced runway prior to data collection. Horses classified as mildly asymmetric or more were excluded from participation (out of five asymmetry degrees: symmetric, very mild asymmetry, mild asymmetry, moderate asymmetry, and severe asymmetry).^14^ This smartphone application was deemed a valid and time‐efficient method for a standardised selection process. Horses of other breeds were excluded.

### 2.2 Study preparation

#### 2.2.1 Surface electromyography and electrode placement

The horses were randomly assigned to one of four groups (*n* = 4 per group): left front, right front, left hind, and right hind. Five muscles of interest were identified in both fore‐ and hindlimb regions by palpating distinct anatomical landmarks while the horse stood square (Table S1).^15^ Small areas of skin over the superficial muscle of interest were clipped (1 mm hair length), and cleaned and degreased with ethanol (80%).

Three electrodes (22 × 28 mm, BlueSensor N‐00‐S, Ambu Medicotest A/S) were placed over the muscles of interest in such a way that they aligned parallel with the expected muscle fibre direction. Regarding the frontlimb region, the muscles of interest were the Splenius muscle, Triceps brachii caput longus muscle, Longissimus dorsi muscle, Extensor digitorum communis muscle, and Ulnaris lateralis muscle. For the hindlimb region, the muscles of interest were the Gluteus medius muscle, Biceps femoris muscle, Semitendinosus muscle, Extensor digitorum longus muscle, and Flexor digitorum profundus muscle. The first electrode was placed in the centre of the region of interest, with the second and third electrodes placed adjacent to the central electrode, resulting in an interelectrode distance of (not smaller than) 22 mm. The true distance is not confirmed after placement, as a negligible effect is to be expected in this repeated‐measures study design. The electrodes were secured with tree resin‐based glue (Superstar Mastix, Kryolan) and with pieces of adhesive foam bandage (Animal Polster, Snögg, Norway).

The reference electrode was placed over the ipsilateral tuber coxae of the measured side. Electromyographic signals were recorded via wire and amplified to a wireless interface (SAGA 32+, TMS International BV), which was secured with a standard girth. Analogue‐digital conversion was performed at a frequency of 2000 samples per second. A counterweight was attached to the contralateral side of the interface to avoid unbalanced pull on the girth, and cables were taped and secured with an adhesive foam bandage to minimise movement artefacts. An illustration of the equipment setup is provided in Figure S1.

#### 2.2.2 Motion tracking

Kinematic data were collected using 10 inertial measurement units (IMUs; ProMove mini, Inertia Technology) using EquiMoves software, placed on the horse's poll, withers, tuber sacralia, tubera coxae, on the lateral sides of all four cannon bones, and on the EMG interface. [Correction added on 12 January 2026, after first online publication: The preceding sentence was corrected.] The IMUs recorded signals at a frequency of 200 samples per second.

### 2.3 Data collection

EMG data were collected during the execution of the four flexion tests, thus while the limb was being flexed (i.e., static) and during the subsequent trot‐up (i.e., dynamic) to investigate muscle activation responses. Before the flexion tests were performed, one baseline measurement of the static condition was conducted, while the horse stood square and still for 60 s. Kinematic data were only collected during trotting (i.e., dynamic). For this we used a 30‐m straight and hard surfaced runway. The horse was walked in‐hand up and down the runway twice after the static baseline measurement to become familiarised with the equipment. Subsequently, the dynamic baseline measurement was collected during trotting up and down the runway twice in‐hand.

Each horse underwent one full‐limb flexion test per leg, performed for 60 s by the same veterinarian (F.M.S.B.). Pilot measurements showed that repeating flexion tests were too extensive for the horse's tolerability. The order of the four flexion tests was determined using an online randomisation tool. The coffin, pastern, fetlock, carpal, and elbow joints were flexed during the full forelimb flexion test. The coffin, pastern, fetlock, stifle, and hip joints were flexed during the full hindlimb flexion test. The horses were trotted in‐hand immediately 2 × 30 m after each of the four flexion tests. The enforced posture of the horse during a full fore‐ and hindlimb flexion test is supplemented in Figure S2.

A 60‐s in‐hand walking period was implemented between flexion tests as ‘wash‐out’ to minimise crossover effects. If the horse moved in a way that required the flexion test to be interrupted, the test was repeated after a 60‐s walking period.

### 2.4 Data analysis

#### 2.4.1 Electromyography

EMG data were analysed using custom‐written MATLAB scripts (The MathWorks). Bipolar EMG signals were derived from the two most proximal unipolar leads, while the most distal lead was used as a backup in case of visual distortions because of losing an electrode or substantial movement artefact. All EMG data were processed with a bidirectional, fourth‐order Butterworth band‐pass filter (40–450 Hz), rectification, and subsequently a bidirectional, second‐order Butterworth low‐pass filter set at 25 Hz to obtain the linear envelope of the signal.^16^ The median peak value from the linear envelope during the in‐hand trotting baseline measurement was used for amplitude normalisation of the signal at each bipolar EMG signal in both the static and dynamic measurements. Five conditions were analysed: baseline, and flexion tests on the left forelimb, right forelimb, left hindlimb, and the right hindlimb. Start and stop times for the flexion tests were manually stored by pressing a trigger button on the EMG interface and processed as an additional input channel.

Kinematic and EMG recordings were synchronised using the three‐dimensional accelerometer data from the EMG interface, the IMU placed on top of the EMG interface, and a cross‐correlation algorithm based on the Euclidian norm. Individual strides during trotting after the flexion tests were defined using a stride detection algorithm.^17^ A stride cycle was defined as the interval from the hoof impact of the hindlimb to the next hoof impact of the same hindlimb on the ipsilateral side of the measured EMG data. Based on footfall timings, EMG data from the first 4 strides of trot were extracted manually, confirmed visually with video images of the measurements. Each stride was time‐normalised to 500 samples (0.2%–100% of the stride cycle). The data were side‐normalised and relabelled as “ipsilateral” (e.g., if the flexion test was applied to the left limb, while EMG data were also collected from the left side) or “contralateral” (e.g., if the flexion test was applied to the right limb, while EMG data were still being collected from the left side).

For analyses of the static part during the flexion test, the median EMG amplitude was calculated between 5 and 55 s after the start of the baseline measurement and the start of a flexion test, for each horse, condition, and muscle. The start of this interval was arbitrarily decided during data analyses to minimise movement artefacts at the beginning of the flexion test. For analyses of the dynamic part subsequent to the static part of the flexion tests, the median normalised EMG amplitude was calculated for each sample across all strides for each horse, condition, and muscle. The amplitude‐normalised EMG data were expressed as a percentage of the median peak during the straight‐line trot.

#### 2.4.2 Locomotion symmetry

Upper‐body vertical displacement asymmetry variables were calculated using proprietary algorithms (Equi‐Pro, Inertia Technology), collected at three segments: poll, withers, and sacrum. [Correction added on 12 January 2026, after first online publication: The preceding sentence was corrected.] Two locomotion asymmetry parameters were calculated per segment. These calculations were based on the double sinusoidal waveform in vertical displacement on a stride‐by‐stride basis. The parameters were derived from the first passage of the 30‐m straight line at the baseline measurement, or after a flexion test was performed. The two parameters were the mean difference in vertical minima (*MinDiff* of the poll, withers, and sacrum), and maxima (*MaxDiff* of the poll, withers, and sacrum). Side normalisation was desired to compare the left‐sided flexion test results with the right‐sided flexion test results, while abiding by the horse's baseline variation. Therefore, the kinematic data set was split into two datasets: one for the left‐sided flexion tests, and one for the right‐sided flexion tests, both including the same baseline results. Then, the results of the left‐sided data sets were multiplied by minus one. As a result of this procedure, positive values indicated ipsilateral‐sided asymmetry, while negative values indicated contralateral‐sided asymmetry concerning the flexed limb, and a value of zero indicated perfect symmetry.

### 2.5 Data analysis

Static EMG data were analysed using statistical analysis software (SPSS version 28.0.1.0). A one‐way repeated measures ANOVA was performed to assess the effect of median muscle activity between flexion tests and baseline per muscle.

Dynamic EMG data were analysed using statistical parametric mapping (SPM, Matlab‐toolbox spm1d version M.0.4.10), to analyse differences in muscle activity between flexion tests and muscles.^18^ The mean stride cycle (0.2%–100%) was used as input per muscle, per condition, per horse. A two‐way repeated measures ANOVA for one‐dimensional measures was used to test for differences between conditions and muscles for the forelimb and hindlimb groups individually, with condition and muscle as factors and normalised muscle activity as the dependent variable. An *F*‐ratio was calculated for each percentage of the stride cycle and compared against a critical *F*‐ratio (F*) with an alpha of 0.05.

Kinematic data were analysed using a linear mixed‐effect model (R‐studio version 4.3.2; packages *nlme* and *emmeans*) to evaluate the effect of the individual flexion tests, relative to each test's own baseline condition, on the MaxDiff and MinDiff of the poll, withers, and pelvis segments separately. The model included flexion test and measured side as fixed effects, with their interaction term, and horse as a random effect. In all statistical analyses, the alpha was set at 0.05, and group averages are presented as estimated marginal means with a 95% confidence interval. Post hoc pairwise comparisons were performed when significant main effects were identified, using a Bonferroni correction.

**FIGURE 1:**
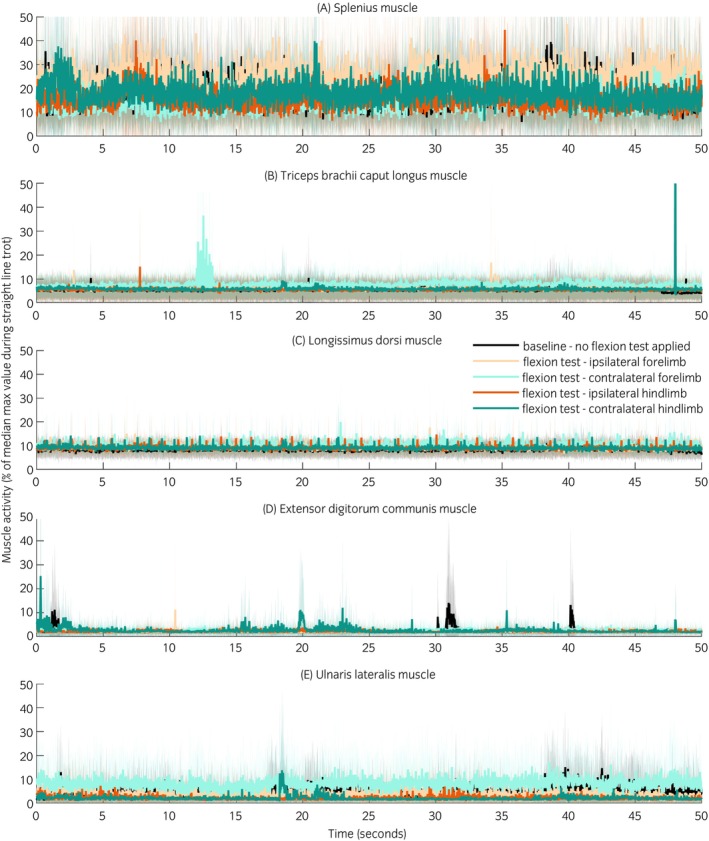
Group averaged muscle activity (*n* = 8) of the (A) Splenius muscle, (B) Triceps brachii muscle, (C) Longissimus dorsi muscle, (D) Extensor digitorum communis muscle and (E) Ulnaris lateralis muscle during four different or no full‐limb flexion tests. The *solid lines* represent the group averaged muscle activity (expressed in percentage of the median max value during straight line trot) per hind side muscle over the first 50 s after the start of applying a flexion test or baseline. *Semi‐transparent coloured areas* represent plus/minus one standard deviation of the group averaged muscle activity.

## 3 RESULTS

### 3.1 Horse characteristics

In total, 39 horses were screened for participation, of which 16 horses were included. Twelve mares and four geldings, with a mean age of 9.9 years (range of 5–22 years), a mean height of 169 cm (range 160–185 cm), were in regular work, used for recreational and professional riding, and housed in boxes with daily turnout.

### 3.2 Static electromyographic data

**FIGURE 2:**
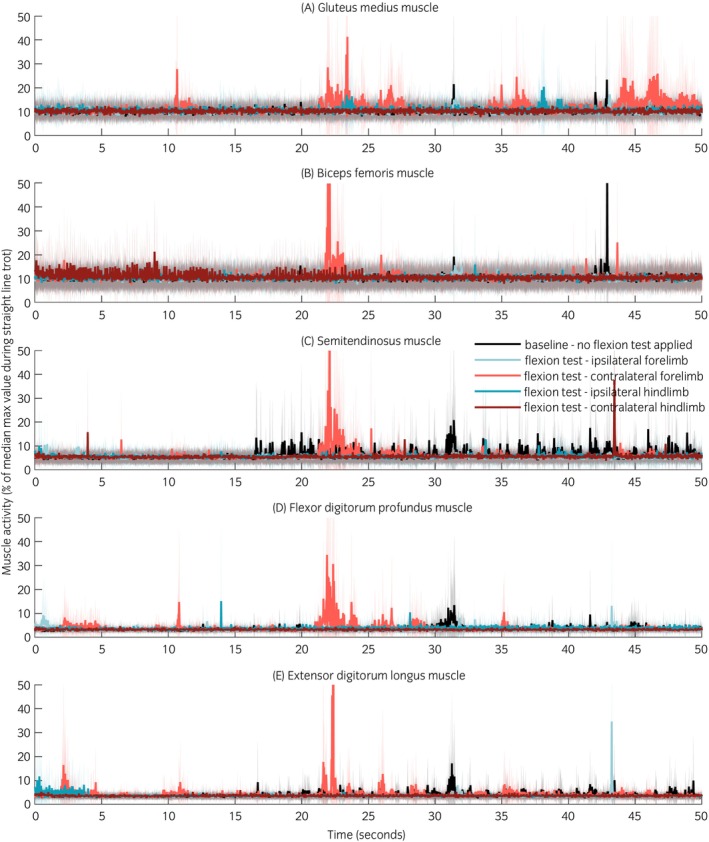
Group averaged muscle activity (*n* = 8) of the (A) Gluteus medius muscle, (B) Semitendinosus muscle, (C) Biceps femoris muscle, (D) Flexor digitorum profundus muscle and (E) Extensor digitorum longus muscle during four different or no full‐limb flexion tests. The *solid lines* represent the group averaged muscle activity (expressed in percentage of the median max value during straight line trot) per hind side muscle over the first 50 s after the start of applying a flexion test or baseline. *Semi‐transparent coloured areas* represent plus/minus one standard deviation of the group averaged muscle activity.

The back‐up bipolar electrode configuration was used in only two of 80 leads because of visual distortions in the original bipolar electrode configuration. No significant differences were observed in normalised muscle activity of the fore‐ or hindlimbs during the static phase of any of the full‐limb flexion tests compared to baseline (*p* = 0.514 and *p* > 0.9, respectively). The grouped averaged results are reported in Table 1, and Figures 1 and 2 illustrate group averaged normalised muscle activity over time of the baseline measurement and when the limb was in a flexed position.

**TABLE 1:** The group averaged muscle activity of the fore‐ (*n* = 8) and hindlimb region (*n* = 8) muscles during seconds 5–50 for the baseline measurement and from the start of a full‐limb flexion test.

Normalised muscle activity	Baseline—no flexion test applied	Flexion test—ipsilateral forelimb	Flexion test—contralateral forelimb	Flexion test—ipsilateral hindlimb	Flexion test—contralateral hindlimb	*p*
Forelimb region muscles						
Splenius muscle	15.7 (9.1 to 22.4)	20.0 (10.7 to 29.2)	12.6 (5.0to20.1)	13.4 (6.7to20.1)	15.6 (9.1to22.0)	0.503
Triceps brachii muscle	5.5 (1.8 to 9.3)	5.9 (1.7 to 10.0)	7.5 (3.2 to 11.8)	5.4 (1.7 to 9.1)	5.6 (1.9 to 9.4)	0.899
Longissimus dorsi muscle	8.4 (5.6 to 11.3)	8.9 (6.6 to 11.1)	9.8 (8.4 to 11.2)	8.8 (6.7 to 10.9)	8.9 (7.0 to 10.8)	0.875
Extensor digitorum communis muscle	1.9 (1.3 to 2.4)	1.8 (1.3 to 2.3)	2.7 (0.5 to 4.8)	2.0 (1.3 to 2.6)	2.0 (1.3 to 2.7)	0.720
Ulnaris lateralis muscle	3.3 (0.1 to 6.4)	2.9 (−0.1 to 5.9)	7.6 (−0.2 to 15.5)	1.8 (0.9 to 2.6)	1.7 (1.1 to 2.2)	0.109
Hindlimb region muscles						
Gluteus medius muscle	10.1 (7.2 to 13.0)	10.3 (7.2 to 13.3)	10.5 (7.5 to 13.5)	10.6 (7.1 to 14.0)	10.2 (7.3 to 13.2)	0.999
Semitendinosus muscle	10.4 (6.2 to 14.6)	10.4 (6.2 to 14.7)	10.5 (6.3 to 14.7)	10.4 (6.2 to 14.6)	10.5 (6.2 to 14.7)	1.000
Biceps femoris muscle	5.3 (3.1 to 7.4)	5.2 (2.9 to 7.5)	5.3 (3.1 to 7.5)	5.5 (2.7 to 8.2)	5.4 (3.2 to 7.7)	1.000
Flexor digitorum profundus muscle	3.0 (2.6 to 3.3)	2.8 (2.4 to 3.2)	3.2 (2.9 to 3.6)	3.5 (2.5 to 4.4)	3.1 (2.3 to 4.0)	0.513
Extensor digitorum longus muscle	3.4 (2.5 to 4.4)	3.4 (2.5 to 4.2)	3.6 (2.6 to 4.6)	3.5 (2.5 to 4.5)	3.5 (2.4 to 4.5)	0.994

*Note*: Applied flexion tests are categorised as applied on the ipsilateral or contralateral side referred to the limb measured with EMG. Muscle activity is reported as normalised muscle activity (percentage of the median peak during a straight‐line trot), as mean values and corresponding 95% confidence interval between brackets.

**FIGURE 3:**
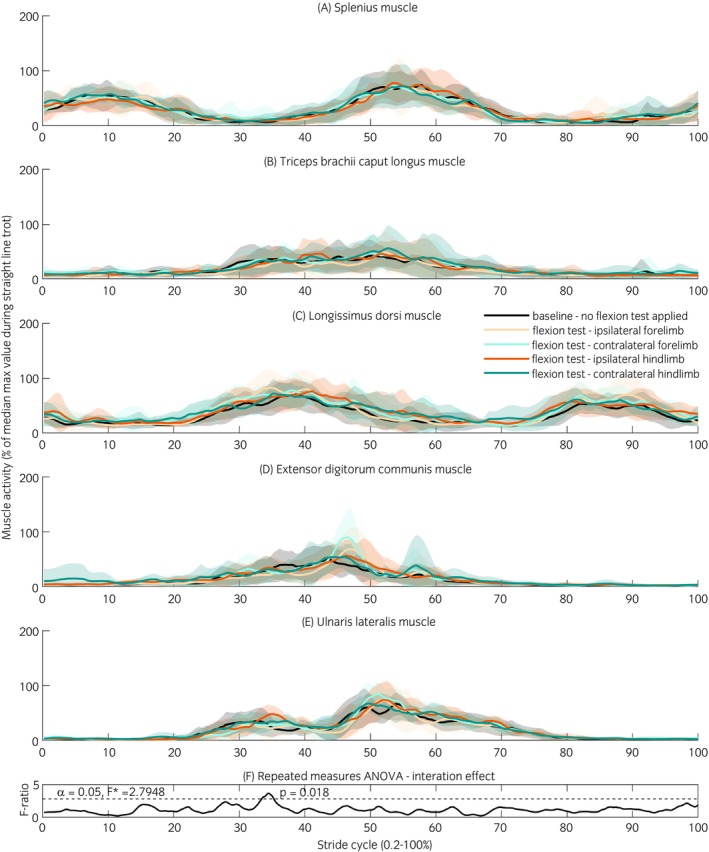
Group averaged muscle activity (*n* = 8) of the (A) Splenius muscle, (B) Triceps brachii muscle, (C) Longissimus dorsi muscle, (D) Extensor digitorum communis muscle and (E) Ulnaris lateralis muscle after four different or no full‐limb flexion tests. The *solid lines* represent the group averaged muscle activity (expressed in percentage of the median max value during straight line trot) per forelimb region muscle per percentage over a stride cycle during trot. *Semi‐transparent coloured areas* represent plus/minus one standard deviation of the group averaged muscle activity. (F) The solid black line is the *F*‐ratio per percentage, determined by a repeated measures ANOVA. The dashed horizontal line represents the critical *F*‐ratio (F*).

### 3.3 Dynamic electromyographic data

**FIGURE 4:**
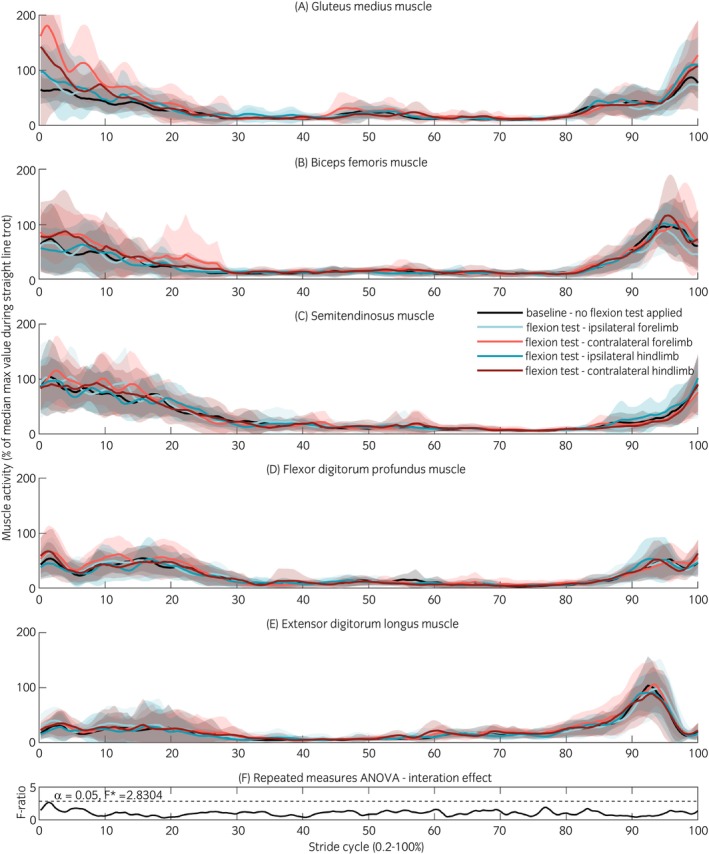
Group averaged muscle activity (*n* = 8) of the (A) Gluteus medius muscle, (B) Semitendinosus muscle, (C) Biceps femoris muscle, (D) Flexor digitorum profundus muscle and (E) Extensor digitorum longus muscle after four different or no full‐limb flexion tests. The *solid lines* represent the group averaged muscle activity (expressed in percentage of the median max value during straight line trot) per hindlimb region muscle per percentage over a stride cycle during trot. *Semi‐transparent coloured areas* represent plus/minus one standard deviation of the group averaged muscle activity. (F) The solid black line is the *F*‐ratio per percentage, determined by a repeated measures ANOVA. The dashed horizontal line represents the critical *F*‐ratio (F*).

In the forelimb region muscles, there was a significant effect of the flexion test on the amplitude of normalised muscle activity between conditions, at mid‐protraction of the forelimb between 33.2 and 35.1 percent of the stride cycle during trot (degrees of freedom [df] (16,112) *F* = 2.79, *p* < 0.05). Post hoc tests revealed no statistically significant effects between individual conditions, when pairwise comparisons were performed within each muscle individually. In the hindlimb region muscles, there was no significant effect of the flexion test on the amplitude of normalised muscle activity between conditions (df(16,112) *F* = 2.83, *p* > 0.05). The group averaged results for fore‐ and hindlimb region muscles are visualised in Figures 3 and 4, respectively.

**FIGURE 5:**
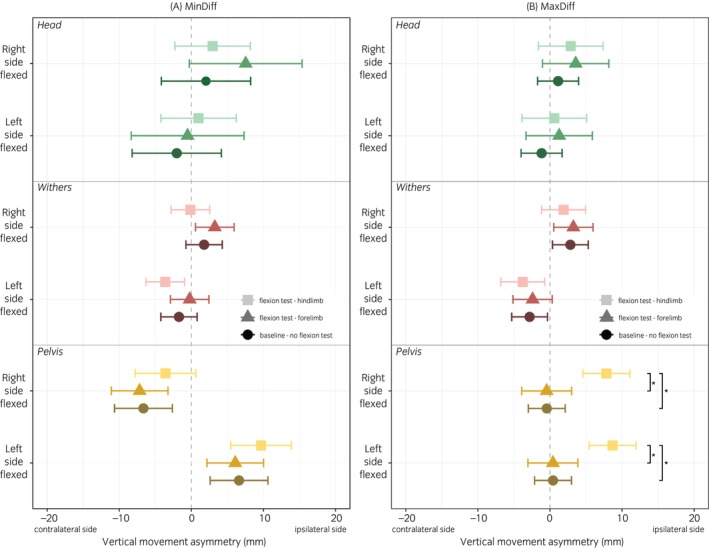
Group averaged (*n* = 16) vertical displacement asymmetry parameters during a straight‐line trot at baseline, or after a full‐limb flexion test of the head, withers, and pelvis segment. (A) MinDiff: mean difference in minimum individual segment position during ipsilateral and contralateral stance phases for each stride during a straight‐line trot. (B) MaxDiff: mean difference in maximal individual segment position during ipsilateral and contralateral stance phases for each stride during a straight‐line trot. Positive values indicate an asymmetry towards the ipsilateral side being flexed, while negative values indicate an asymmetry towards the contralateral side being flexed. Group average results are reported in millimetres, as mean values and corresponding 95% confidence interval between brackets. mm, millimetres; *, post hoc pairwise comparisons *p* < 0.05.

### 3.4 Dynamic kinematic data

The group averaged results are reported in Table 2 and Figure 5. Statistically significant differences were observed in MaxDiff of the pelvis segment between trotting conditions (*p* = 0.001).

**TABLE 2:** The group averaged (*n* = 16) vertical displacement outcome parameters during a straight‐line trot at baseline, or after a full‐limb flexion test.

Vertical displacement	Baseline—no flexion test applied (modified)	Flexion test applied to left forelimb	Flexion test applied to left hindlimb	Baseline—no flexion test applied (original)	Flexion test applied to right forelimb	Flexion test applied to right hindlimb	*p*
MinDiff							
Poll	−2.0 (−8.2 to 4.2)	−0.5 (−8.3 to 7.3)	1.0 (−4.2 to 6.2)	2.0 (−4.2 to 8.2)	7.5 (−0.3 to 15.4)	2.9 (−2.3 to 8.2)	0.549
Withers	−1.7 (−4.8 to 1.4)	−0.1 (−3.5 to 3.2)	−3.7 (−7.1 to −0.4)	1.7 (−1.4 to 4.8)	3.1 (−0.2 to 6.4)	−0.1 (−3.4 to 3.3)	0.098
Sacrum	6.2 (1.3 to 11.2)	6.5 (1.7 to 11.3)	9.7 (4.5 to 15.0)	−6.2 (−11.2 to −1.3)	−7.5 (−12.3 to −2.7)	−3.6 (−8.9 to 1.7)	0.274
MaxDiff							
Poll	−1.6 (−4.8 to 1.6)	2.0 (−4.1 to 8.1)	1.6 (−4.3 to 7.5)	1.6 (−1.6 to 4.8)	2.9 (−3.2 to 8.9)	1.9 (−4.0 to 7.8)	0.474
Withers	−3.5 (−6.5 to −0.6)	−2.2 (−5.6 to 1.2)	−2.9 (−6.8 to 1.0)	3.5 (0.6 to 6.5)	3.0 (−0.4 to 6.4)	1.0 (−2.9 to 4.9)	0.719
Sacrum	0.4 (−2.6 to 3.3)	0.5 (−4.0 to 5.0)	8.8 (4.6 to 12.9)	−0.4 (−3.3 to 2.6)	−0.5 (−5.0 to 3.9)	7.7 (3.6 to 11.9)	0.001

*Note*: MinDiff; mean difference in minimum individual segment position during ipsilateral and contralateral stance phases for each stride during a straight‐line trot. MaxDiff; mean difference in maximal individual segment position during ipsilateral and contralateral stance phases for each stride during a straight‐line trot. Positive values indicate an asymmetry towards the ipsilateral side being flexed, while negative values indicate asymmetry towards the contralateral side being flexed. Group averaged results are reported in millimetres, as mean values and corresponding 95% confidence interval between brackets.

In the left hindlimb, the between group difference in MaxDiff between trotting after a left hindlimb flexion test and baseline trotting was 8.4 mm (±2.4 standard error [SE], *p* = 0.002), and between trotting after a left hindlimb flexion test and trotting after a left forelimb flexion test was 8.3 mm (±2.9 SE, *p* = 0.008).

In the right hindlimb, the between group difference in MaxDiff vertical displacement between trotting after a right hindlimb flexion test and baseline trotting was 8.1 mm (±2.4 SE, *p* = 0.004), and between trotting after a right hindlimb flexion test and trotting after a right forelimb flexion test was 8.3 mm (±2.9 SE, *p* = 0.008). Other effects of full‐limb flexion tests on vertical displacement parameters during trotting were nonsignificant.

## 4 DISCUSSION

In warmblood horses with no or very mild asymmetry, a full‐limb flexion test did not have an effect on the muscle activity of the studied muscles during the application of a flexion test, nor during trotting in a straight line immediately after. The pelvis was pushed up significantly more during the suspension phase after the ipsilateral hindlimb push‐off (with regard to the flexed limb) during trotting after a full‐limb flexion test of both the left and right hindlimbs, compared to baseline trotting and trotting after full‐limb flexion tests of the forelimbs. Based on these findings, our hypothesis is partially rejected: while changes in locomotion asymmetry were observed, the expected alterations in muscle activity were not detected.

### 4.1 Compared to literature

#### 4.1.1 Static electromyographic data

There are currently no studies that have examined the effects of flexion tests on muscle activity. The method proposed for measuring superficial fore‐ and hindlimb region muscle activity during flexion tests was conducted for the first time. During a full‐limb flexion test, redistribution of the horse's centre of gravity and repositioning of the contralateral supporting limb were expected, activating certain muscles continuously. Since no fatiguing component was expected from the 1‐min effort, the entire duration of the flexion test was included in the data analysis, rather than analysing only a small epoch at the end.

Although the horse's position was maintained as still as possible, some movements in the neck and head were inevitable. A median value over the whole static part was therefore used to cope with outliers, instead of the mean value.^19^ No changes in muscle activity were observed while the fore‐ or hindlimbs were held in the flexed position. Amplitude normalisation against trotting in a straight line may overestimate normalised muscle activity levels, particularly if the Splenius muscle is relatively less active during trotting; therefore, the true percentage of activity is speculative. A median value was chosen instead of a mean or maximal value for amplitude normalisation, as recommended in the literature.^20^ Our approach helps account for outliers caused by unrelated movements during trotting and estimates a robust normalisation value. An example supporting this decision is visualised in Figures S3 and S4. The absence of a significant effect on muscle activity suggests that maintaining balance while standing still on three limbs is a relatively passive process for the muscles included, requiring minimal muscular activity. Due to the lack of comparative literature, these results cannot be directly compared with findings from other studies.

#### 4.1.2 Dynamic electromyographic data

There were no differences in muscle activity of the Splenius muscle, Triceps brachii muscle, Longissimus dorsi muscle, Extensor digitorum communis muscle, Ulnaris lateralis muscle, Gluteus medius muscle, Biceps femoris muscle, Semitendinosus muscle, Extensor digitorum communis muscle, and Flexor digitorum profundus muscle between baseline trot and the four full‐limb flexion tests of the fore‐ or hindlimbs in our study. There are no clear indications from the EMG data to explain the statistically significant effect observed on pelvic MaxDiff during trotting after a hindlimb flexion test.

Previous reports on the effects of induced fore‐ or hindlimb lameness on muscle activity have shown both increases and decreases in multiple appendicular muscles in the fore‐ or hindlimbs, or spinal muscles during trotting.^11^, ^12^ The suggested temporary localised pain stimuli by a flexion test is likely to be provoking most likely a smaller degree of discomfort than induced lameness (e.g., screw/bolt under a horseshoe), as reported MaxDiff values of the sacrum are larger in these studies (on average between 27.9 and 31.3 mm).^11^, ^12^

#### 4.1.3 Dynamic kinematic data

Applying a hindlimb flexion test resulted in a confirmed effect on pelvic kinematics during subsequent trotting. The pelvic segment showed greater upward displacement (MaxDiff) during the suspension phase of the ipsilateral hindlimb compared to both baseline trotting and following the application of a forelimb flexion test. This effect was observed in both the left and right hindlimb, confirming the presence of a consistent response. These findings align with previous studies investigating the impact of hindlimb flexion tests on pelvic MaxDiff.^9^, ^10^

The absence of an effect on the pelvic MinDiff vertical displacement parameter is consistent with the aforementioned studies.^9^, ^10^ No contralateral asymmetry was observed in vertical displacement of the withers segment, a compensatory strategy typically seen in cases of induced or naturally occurring hindlimb lameness.^21^, ^22^ This absence suggests that hindlimb flexion tests may affect different anatomical structures than those involved in lameness, thereby provoking a distinct compensatory response. It remains unclear to what extent the involved joints were flexed during the full‐limb flexion tests. Computed musculoskeletal horse models could help in simulating a revived version of a flexion test with all joints flexed maximally.^23^

The observed effects on kinematic parameters may have been underestimated, as individual horses appear to respond differently to flexion tests, distinguishing between responders and nonresponders. The rate of positive responses is known to increase with greater force and longer duration of flexion, particularly in the forelimbs.^24^ In our study, duration was standardised and considered adequate. Applied force was not quantified and may have varied between horses. However, they were all performed by the same veterinarian, and this was deemed sufficiently consistent.^25^ Such variability, together with the large spread in certain kinematic variables (e.g., MinDiff of the head segment after a forelimb test), likely explains why measurable effects were seen in some horses but not others (possible responders and nonresponders). Additional factors such as age and sex may also contribute to the heterogeneous responses observed in our cohort, although these effects had previously been subjectively measured in a large homogeneous group of Dutch Warmblood horses.^26^

#### 4.1.4 Movement asymmetry in owner‐sound horses

In this study, owner‐sound horses were selected for data collection after confirmation of a symmetric movement pattern during a straight‐line trot with the Sleip gait analysis mobile phone application.^14^ Out of 39 horses initially assessed, only 22 (56%) were deemed sound for participation. This finding is in line with the findings of other studies, recruiting owner‐sound horses.^27^, ^28^, ^29^ In one study, 73% (*n* = 161 of 222) of the owner‐sound horses showed significant movement asymmetries during a straight‐line trot.^29^ It is unknown in both studies if the asymmetric movement pattern was related to pain or to a natural variation. The investigated cohort of horses also showed to some extent an asymmetrical movement pattern at baseline (Table 2).

### 4.2 Clinical implications

An effect on the MaxDiff component of the pelvis following a hindlimb flexion test can be expected. Based on our results, this effect is not attributable to muscular activity of the measured muscles. Instead, it suggests that the flexion test may trigger or activate another structure in the hindlimb. Whether this originates from ligaments, joints or tendons remains unclear. The presence of subclinical pain cannot be excluded, even though the study population was considered sound by their owners. Therefore, the use of this test should be reconsidered and approached with caution, regardless of the intended application (e.g., lameness assessment or pre‐purchase evaluations).

### 4.3 Limitations

This study has several limitations. First, the effect of only a full‐limb flexion test was investigated. The primary reason for focusing on the full‐limb flexion test is that this type of test flexes the limb's joints the most compared to a distal or carpal/tarsal flexion test. It maximises the potential effect on inducing movement asymmetry and detecting subsequent changes in muscle activity and kinematic parameters. Moreover, no effect on the other flexion tests should be expected if a full‐limb flexion test is negative. Conversely, if the full‐limb flexion test had indeed shown relevant effects, then extra research would have been meaningful to further analyse other flexion tests. Another argument was that piloting data suggested no significant differences in muscle activity between a distal‐, carpal/tarsal, or full‐limb flexion test (*n* = 1).

Second, the dynamic kinematic outcome parameters were estimated means for the whole first straight‐line trot after a full‐limb flexion test; however the electromyographic data was extracted from the first four strides of the straight‐line trot. The kinematic parameters cannot explain the dynamic electromyographic data directly, as a possible effect of the flexion tests is suggested to dissipate.^9^, ^10^ A stride‐by‐stride analysis during subsequent trotting could provide more detailed insights into the degree of dissipative effect and the relationship between potential effects on muscle activity and kinematic parameters. This was however not feasible because of the proprietary algorithms used for extraction of kinematic parameters.

Third, although the flexion tests were executed by a single veterinarian to minimise inter‐executor variability, the applied force on the limb during the flexion test was not measured.^25^ We observed that the horse's initial response after the ipsilateral limb was lifted for the flexion test was to redistribute the centre of gravity more over the contralateral limb to maintain stability. To what extent the horse also relied on the veterinarian for maintaining balance, and whether this had a possible effect on the supporting contralateral limb's muscle activity, remains unknown. Furthermore, postural sway during the static phases was not assessed, which could have influenced muscle activity levels in the static phases.

Fourth, speed normalisation was not feasible in this study, making it unknown whether the horses trotted at consistent speeds between conditions, which could have influenced muscle activity amplitudes. However, no substantial visual differences in stride duration between horses across conditions were observed, minimising concerns about potential interference (Figure S5).

Fifth, the wired connection between the EMG electrodes and the interface limited the possibility of bilateral EMG measurements and restricted coverage of both forelimb and hindlimb muscles. Therefore, EMG signals from the left and right sides were measured separately. Horses were tested in random order, and the data were subsequently normalised relative to the ipsilateral and contralateral sides to minimise the effect of motor laterality.^30^

Sixth, it could be argued that postural muscles, such as the abdominal muscles or deeper thoracolumbar and cervical muscles, would also have been appropriate to investigate. However, the 10 muscles selected for this study were chosen because they represent the most relevant superficial muscles involved in locomotion, as indicated by pilot testing, and because their inclusion was compatible with the available equipment capacity.

### 4.4 Conclusion

Our study showed that there was no significant change in the activity of the superficial muscles studied here between baseline and flexion test conditions in sound horses. A full hindlimb flexion test induces an ipsilateral locomotion asymmetry in the pelvis during subsequent trotting, as confirmed by individual results in the application of a left and right full hindlimb flexion test.

## FUNDING INFORMATION

This study is funded by Friends of VetMed.

## CONFLICT OF INTEREST STATEMENT

Filipe M. Serra Bragança is an employee of Sleip AI AB which provides a commercially available diagnostic tool for detecting lameness in horses from a smartphone and which was used in this study.

## AUTHOR CONTRIBUTIONS

**Marijke Jonkhart:** Project administration; methodology; investigation; conceptualization; resources; writing – original draft; validation; software; data curation; formal analysis. **Filipe M. Serra Bragança:** Conceptualization; investigation; funding acquisition; methodology; validation; writing – review and editing; software; project administration; supervision; resources. **Ineke H. Smit:** Conceptualization; methodology; visualization; writing – review and editing; formal analysis. **Harold Brommer:** Conceptualization; methodology; writing – review and editing; supervision; resources. **Jozef J. M. Suskens:** Conceptualization; investigation; writing – original draft; methodology; validation; visualization; software; formal analysis; project administration; data curation; supervision.

## DATA INTEGRITY STATEMENT

Marijke Jonkhart had full access to all the data in the study and takes responsibility for the integrity of the data and the accuracy of data analysis.

## ETHICAL ANIMAL RESEARCH

This research was approved by the institutional welfare committee of Utrecht University (reference number: 10801‐2023‐03).

## INFORMED CONSENT

Participating owners gave written consent for the inclusion of their horses in the study.

## Supporting information


**Figure S1.** Equipment setup.

**Figure S2.** Full‐limb flexion.

**Figure S3.** Example of a median, mean, and max normalisation value.

**Figure S4.** Example of a median, mean, and max normalisation value.

**Figure S5.** Mean stride duration.

**Table S1.** Electrode placement.

## Data Availability

The data that support the findings of this study are openly available in dataverse.nl at https://doi.org/10.34894/AL25AD.
