# Supplementary material for: The effect of full‐limb flexion tests on static and dynamic muscle activity and locomotion asymmetry in owner‐sound horses
Source: Equine Vet J. 2025 Dec 2;58(5):1505–17. doi: 10.1111/evj.70130 (PMC13447879; doi:10.1111/evj.70130)

Figure S4. Histogram distribution of the maximal values in muscle activity of the semitendinosus per stride (n = 37) in the baseline straight line trotting condition of a random subject (H006). The corresponding median maximal value; vertical green line, the corresponding mean maximal value; vertical red line, and the corresponding max maximal value; vertical dotted blue line

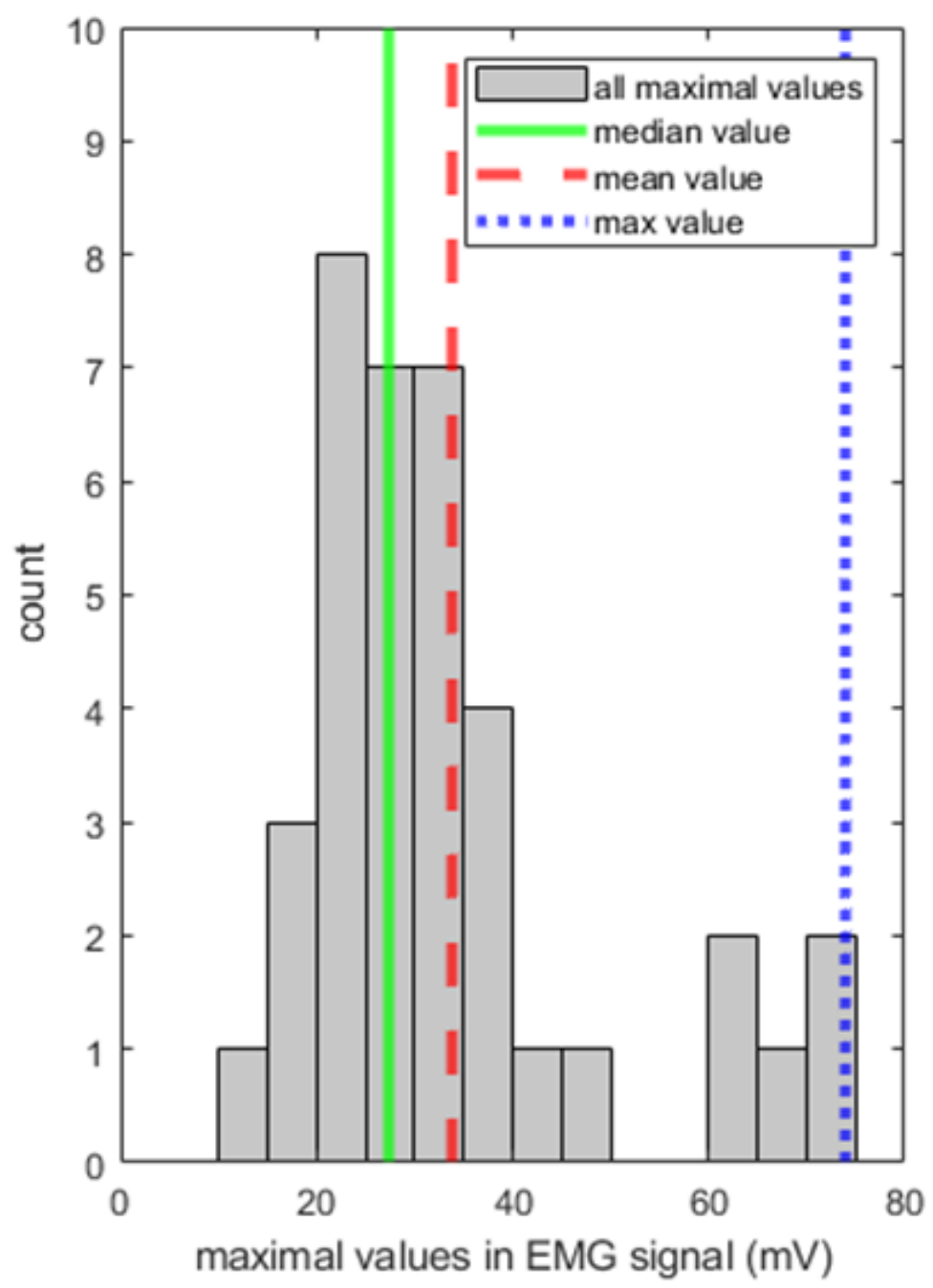

Supplement: Supplementary file 1 [file EVJ-58-1505-s001.pdf]
