# Supplementary material for: The effect of full‐limb flexion tests on static and dynamic muscle activity and locomotion asymmetry in owner‐sound horses
Source: Equine Vet J. 2025 Dec 2;58(5):1505–17. doi: 10.1111/evj.70130 (PMC13447879; doi:10.1111/evj.70130)

Equine Veterinary Journal BEVA

Figure S1. (A) Setup of a horse with equipment for measuring electromyographic data over the right forelimb. Electrodes are placed on the skin over the Splenius muscle, Triceps brachii caput longus muscle, Longissimus dorsi muscle, Extensor digitorum communis muscle, and the Ulnaris lateralis muscle. **(B)** Setup of a horse with equipment for measuring electromyographic data over the right hindlimb. Electrodes are placed on the skin over the Gluteus medius muscle, Biceps femoris muscle, Semitendinosus muscle, Extensor digitorum longus muscle, and the Flexor digitorum profundus muscle. **(A-B)** Inertial measurement units are placed on the horse's poll, withers, tuber sacrale, tubera coxae, and on the lateral sides of the cannon bones.

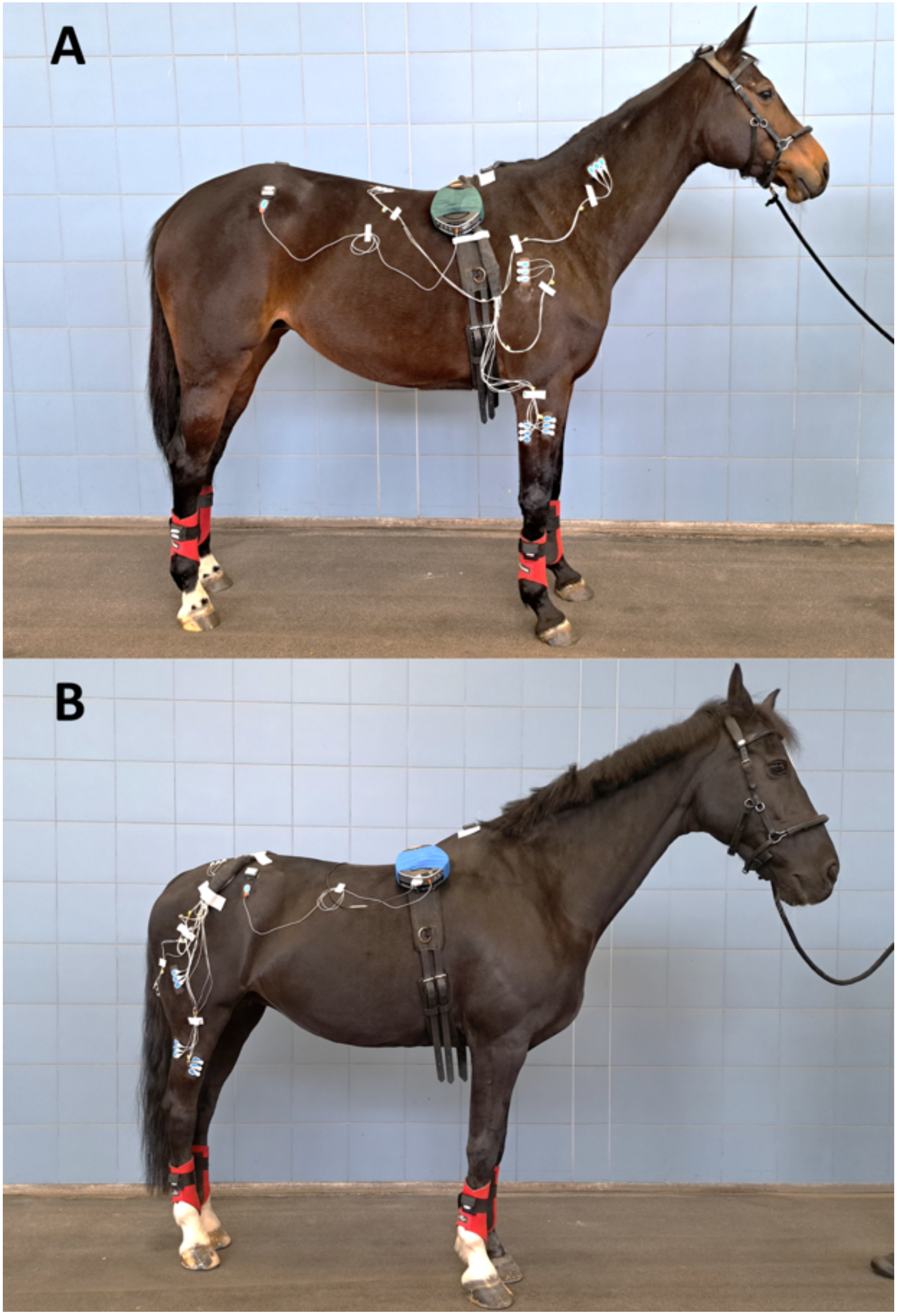

Supplement: Supplementary file 2 [file EVJ-58-1505-s002.pdf]
