# Supplementary material for: The effect of full‐limb flexion tests on static and dynamic muscle activity and locomotion asymmetry in owner‐sound horses
Source: Equine Vet J. 2025 Dec 2;58(5):1505–17. doi: 10.1111/evj.70130 (PMC13447879; doi:10.1111/evj.70130)
Supplement: Supplementary file 4 [file EVJ-58-1505-s004.pdf]

Table S1. Anatomical landmarks used for electrode placement. Optimal electrode locations are presented per measured muscle as the percentage between anatomical landmarks measured from the proximal or cranial landmark to the caudal or distal landmark.

**Muscle**	**Cranial/Proximal landmark**	**Caudal/Distal landmark**	**Additional information**	**Percentage of the line between the anatomical landmarks**
Splenius muscle	Wing of the atlas	Cranial angle of the scapula	-	56.5 – 59.5%
Longissimus dorsi muscle	Top of the withers	Between the tuber sacrale	6 cm out of the midline of the horse (processus spinosus)	45 – 57%
Triceps brachii caput longus muscle	Tip of the spina scapula	Olecranon	-	43.5 – 53.5%
Extensor digitorum communis muscle	Lateral tuberosity of the radius	Lateral styloid process of the radius – ventral side	-	30 – 43.5%
Ulnaris lateralis muscle	Lateral tuberosity of the radius	Lateral styloid process of the radius – caudal side	-	36.5 – 47%
Gluteus medius muscle	Processus spinosus of L4	Tuber ischiadicum	-	32 – 35%
Semitendinosus muscle	Tuber ischiadicum	At height of the insertion of the gracilis muscle	-	27.5 – 50%
Biceps femoris muscle	Caudal part of the greater trochanter of the femur	Lateral tibial condyle and head of the fibula	-	24 – 37%
Extensor digitorum longus muscle	Tibial tuberosity	Lateral ridge of trochlea of talus	-	42 – 50%
Flexor digitorum profundus muscle	Lateral tibial condyle and head of the fibula	Tuber calcanei	Palpation required	26.5 – 68.8%
