# Supplementary material for: The effect of full‐limb flexion tests on static and dynamic muscle activity and locomotion asymmetry in owner‐sound horses
Source: Equine Vet J. 2025 Dec 2;58(5):1505–17. doi: 10.1111/evj.70130 (PMC13447879; doi:10.1111/evj.70130)

Equine Veterinary Journal BEVA

Figure S3. Muscle activity of the semitendinosus per stride (n = 37) in the baseline straight line trotting condition of a random subject (H006). With the maximal values per stride, horizontal grey lines, the corresponding median maximal value; horizontal green line, the corresponding mean maximal value; horizontal dashed red line, the corresponding max maximal value; horizontal dotted blue line.

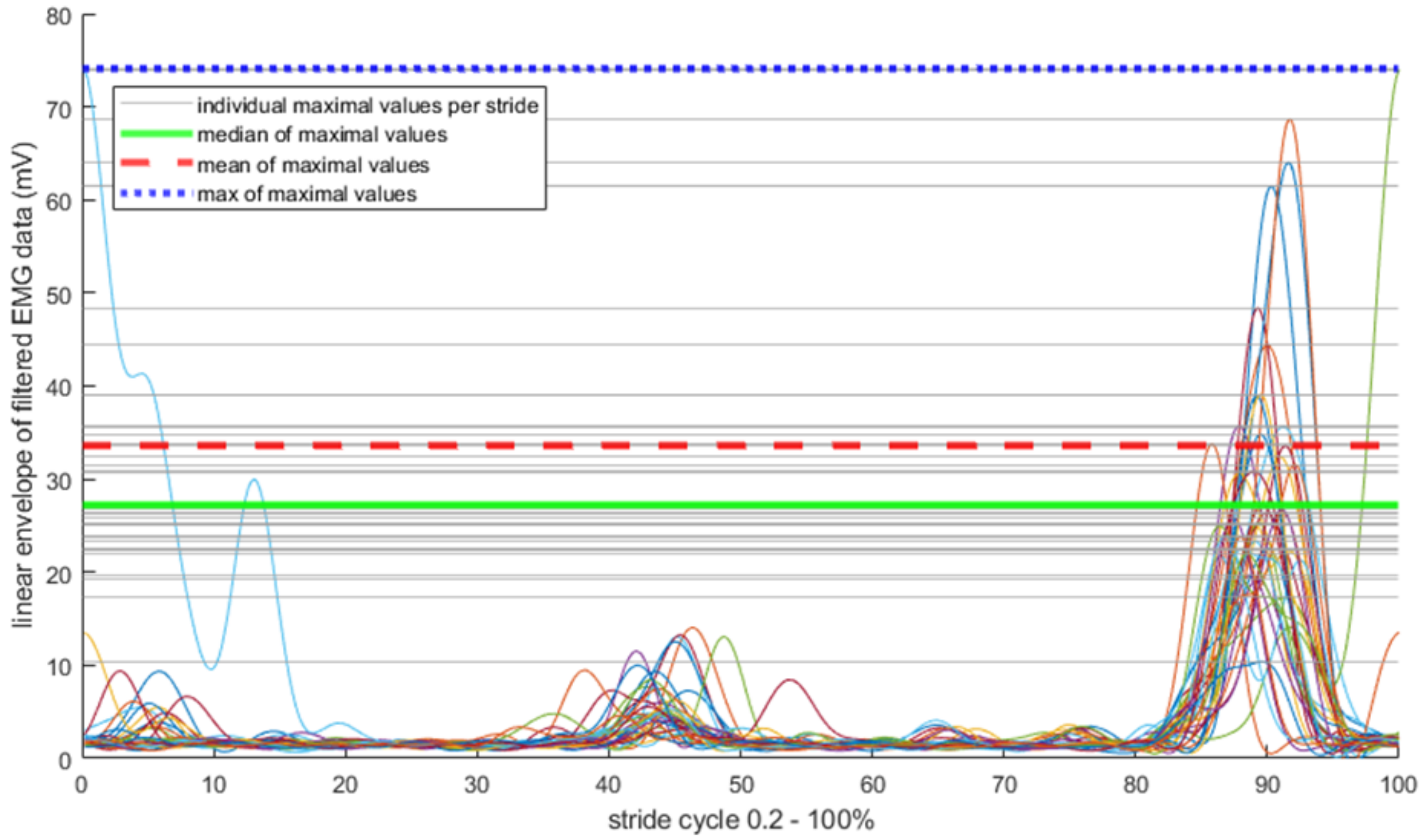

Supplement: Supplementary file 5 [file EVJ-58-1505-s005.pdf]
