# Supplementary material for: The effect of full‐limb flexion tests on static and dynamic muscle activity and locomotion asymmetry in owner‐sound horses
Source: Equine Vet J. 2025 Dec 2;58(5):1505–17. doi: 10.1111/evj.70130 (PMC13447879; doi:10.1111/evj.70130)

Equine Veterinary Journal BEVA

Figure S2. Example of a full limb flexion test applied on **(A)** the right forelimb, and **(B)** the right hindlimb.

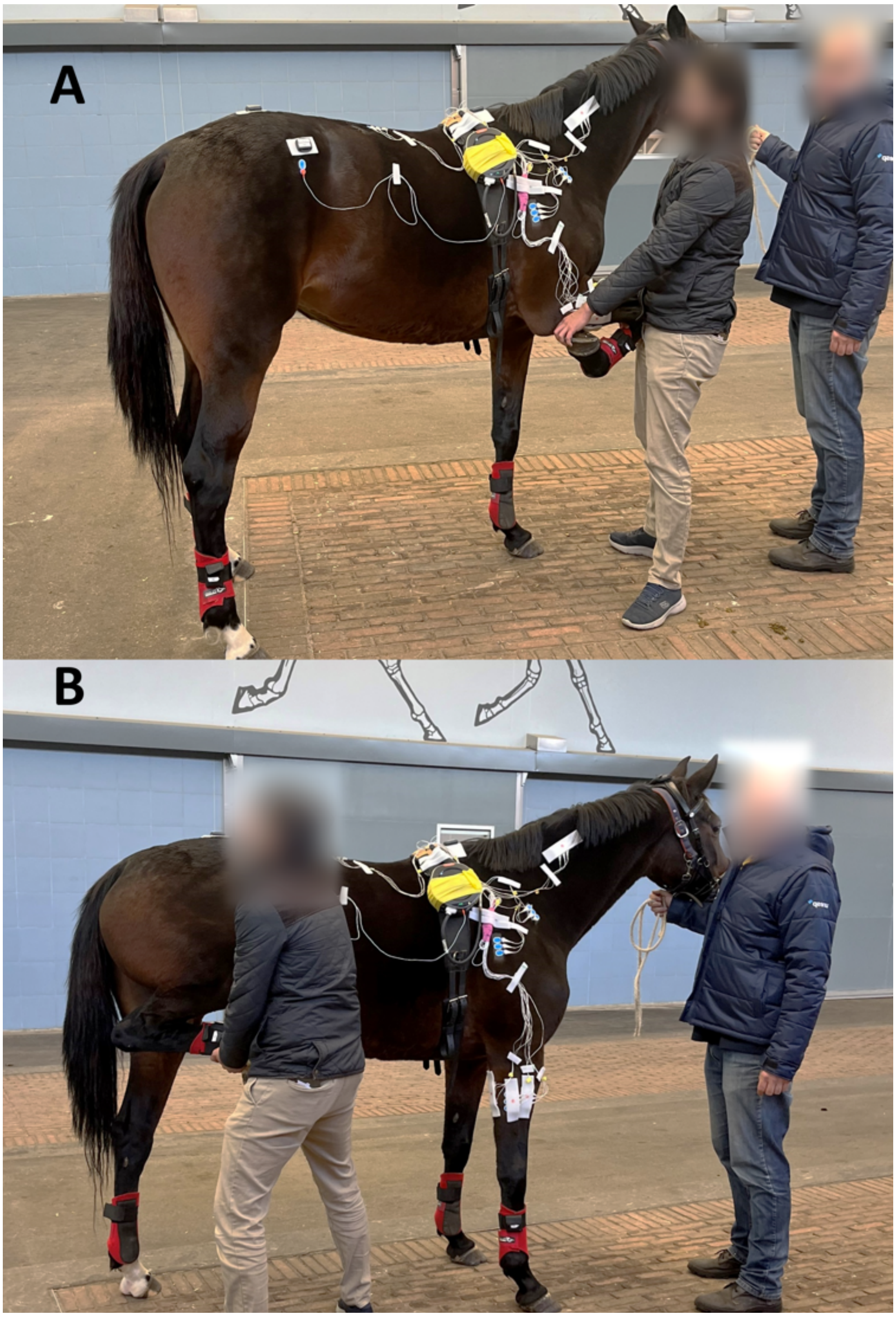

Supplement: Supplementary file 6 [file EVJ-58-1505-s006.pdf]
